# Optimization of a plant regeneration and genetic transformation protocol for *Eucalyptus* clonal genotypes

**DOI:** 10.1186/1753-6561-5-S7-P132

**Published:** 2011-09-13

**Authors:** Raj Deepika, Adri Veale, Cathleen Ma, Steven H Strauss, Alexander A Myburg

**Affiliations:** 1 Department of Genetics, Forestry and Agricultural Biotechnology Institute (FABI), University of Pretoria, Pretoria 0002, South Africa; 2 Department of Forest Ecosystems and Society, 321 Richardson Hall, Oregon State University, Corvallis, OR, 97330, USA

## Background

*Agrobacterium* mediated gene transfer technology offers the potential to introduce novel, high-value traits into selected, elite tree genotypes. Significant progress has been made in the regeneration and genetic transformation of *Eucalyptus* trees, but transformation efficiency has generally been low for eucalypts [1], especially for elite clonal genotypes. As a preliminary step towards the regeneration of explants derived from clonal material and the production of transgenic plants from commercially important *Eucalyptus* genotypes, there is a need to identify genotypes exhibiting high regeneration capacity. In this ongoing study, we are comparing eight *Eucalyptus* genotypes for callus induction and shoot regeneration potential. Browning of callus tissue and surrounding culture media is a common obstacle limiting regeneration of shoots in eucalypts. Hence, a second aim is to minimize the oxidation of phenolic compounds released from the wounded tissue and improve shoot regeneration rates.

## Methods

Shoot buds collected from potted ramets of eight *Eucalyptus* clones (Sappi and Mondi, South Africa) were established under *in vitro* conditionson MS [2] basal medium containing BAP (Benzyl adenine purine). The established clones consisted of four *E. grandis* (G1, G2, G3 and G4), one *E. grandis* x *E. nitens* (GN1), two *E. grandis* x *E. camaldulensis* (GC1 and GC2) and one *E. grandis* x *E. urophylla* (GU1) genotype*.* Leaf explants were excised from *in vitro* shoot cultures and cultured on MS basal medium containing BAP, NAA (Naphthalene acetic acid) and TDZ (Thidiazuron). Browning and necrosis of the callus as well as surrounding culture media was observed in all cultures and PVP (polyvinylpyrrolidone) was therefore added to the media to reduce oxidation.

A standardised regeneration protocol was used for the optimization of *Agrobacterium* mediated genetic transformation of selected *Eucalyptus* clones*.* The *Agrobacterium tumefaciens* strain AGL1 containing pMDC162 with the *uidA* gene (β-glucuronidase; GUS) and *hpt* (hygromycin phosphotransferase) gene was used for transformation. To minimize extensive exudation of phenolic compounds from the wounded explants, the leaves were subjected to different wounding methods. Intact leaves were excised from *in vitro* shoot cultures and preconditioned for 24 hrs on callus induction medium followed by wounding with a surgical blade prior to *Agrobacterium* infection. The leaves were wounded in two different ways: (a) removing the edges of the leaf, making 8-10 small wounds and cutting the leaf transversally into two equal halves and (b) making 5-6 small wounds in the intact leaf without damaging the rest of the leaf. Alternatively, the leaves were wounded prior to 24 h preconditioning using the first wounding method (a). The explants were immersed in a bacterial solution (A_600_ = 0.8) for 1 hr followed by blotting with sterile filter paper. The explants were co-cultivated for 24 hrs and stained for GUS activity after two days to determine the percentage of explants showing transient GUS expression.

## Results and discussion

**Figure 1:**
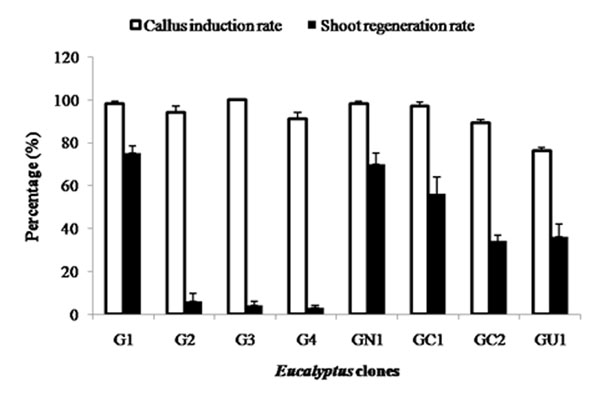
Effect of genotype on percentage of callus induction and shoot regeneration after one month of culture on regeneration media without PVP. Callus induction rate= Number of explants inducing callus / Total number of leaf explants cultured x 100; Shoot regeneration rate= Number of calli inducing shoots/ Total number of leaf explants cultured x 100. Error bars indicate the standard error of the mean of five replicates.

**Figure 2:**
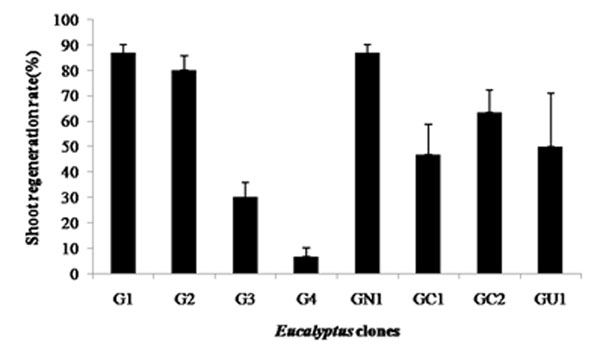
Percentage of leaf explants exhibiting shoot bud induction after one month of culture on shoot regeneration medium containing PVP. Error bars indicate the standard error of the mean of three replicates.

Callus initiation was observed at the cut edges and wounded regions of the leaf explants after 20 days of incubation in complete darkness at 25 ± 2^°^C. Shoot regeneration was also observed on the same medium after 30 days of culture. Callus induction percentages of 76% (GU1) to 100% (G3) and shoot regeneration percentages of 3.4% (G4) to 75% (G1) were obtained from the leaf explants (Figure 1).Tissue oxidation was observed in all of the cultures when exposed to light, which hampered shoot bud development and elongation considerably. Addition of PVP to the media resulted in reduction of tissue browning and increased shoot regeneration rate ranging from 6.7% (G4) to 86.7% (G1 and GN1) (Figure 2). Browning of the tissue and surrounding media occurs due to oxidation of the phenolic compounds by polyphenoloxidase, peroxidise or exposure to air which can be reduced by the addition of antioxidants such as PVP to the culture media [3]. Based on the modified protocol including the addition of PVP, *Eucalyptus grandis* (G1) and *E. grandis* x *E. nitens* (GN1) exhibited the highest shoot regeneration potential (86.7%) and may serve as suitable starting material for further genetic transformation studies.

Different wounding methods did not have a significant effect on transient GUS expression in leaf explants. However, good GUS activity was detected in the transformed callus induced from the leaf explants wounded prior to preconditioning.

## Conclusion

We have achieved efficient and rapid callus induction, as well as shoot regeneration for selected *Eucalyptus* clones. The information presented here forms the basis for ongoing optimization of a protocol for the generation of transgenic plants of clonal *Eucalyptus* genotypes.
